# Reply to the “Criticism” of Dr. H. W. Boyce in Relation to an Article by the Writer upon “Cases in Practice, with Remarks on the Use of Veratrum Viride”

**Published:** 1871-06

**Authors:** B. O. Reynolds

**Affiliations:** Geneva, Wis.


ARTICLE III.—*Reply to the "Criticism" of Dr. H. W. Boyce in Relation to an Article by the Writer upon "Cases in Practice, with Remarks on the Use of Veratrum Viride."* By B. O. REYNOLDS, M.D., Geneva, Wis.

The doctor opens by saying: "The report of cases, to be worth anything to the profession, should set forth all the facts truthfully."

In this statement the writer heartily concurs, as an earnest of which, I introduce the following:

GENEVA, WIS., April 13, 1871.

We certify that the account of Ida's illness and treatment, as published in the February number of *Chicago Medical Journal*, 1871, is strictly accurate and true, to the best of our knowledge and belief; and that the "Criticism" in the April number contains many errors and misrepresentations.

E. L. GILBERT, } Parents.

MRS. B. C. GILBERT, }

MISS A. CURTIS, Nurse.

This effectually disposes of this case in all its details, while it was under my observation, so far as these very intelligent parties were capable of understanding the facts and circumstances detailed in our respective reports. Apply to this the old maxim: "Falsus in uno, falsus in omnibus," and litttle more need be said in relation to the subject; yet I may be pardoned for commenting briefly on some few of the more prominent "errors" contained in the "Criticism," and the first we will notice is this:

"On the second consultation with Dr. R——, it was concluded to try the effects of veratrum viride, if an opportunity should occur," and "it was left with him to carry out the programme." Suppose for a moment this statement to be correct, when would it be expected "an opportunity would occur"? Does he inform us how much veratrum was put down on "the programme"?

The patient had swallowed nothing for many hours, and it was believed by all that she was unable to do so; indeed, she was thought to be dying most of the afternoon and evening, and the doctor expressed himself to all, the writer included, that there "was no hope," and for me "to do anything that I saw fit, or thought advisable"; no medicine was proposed, discussed, or suggested by either of us at that time; the doctor left immediately on my arrival.

No medicine was given her for nearly an hour after my arrival, except the inhalation, which they had found necessary to partially moderate her convulsions. I confess I looked upon the case as almost hopeless, and the only "programme" in our minds, at that time, was the death of our patient within a few hours at most.

The next point that I notice is the doctor's account of her condition next morning. He says: "I examined the patient next morning, but failed to see any marked improvement. Pulse and respirations remained extremely rapid, and there was great restlessness." He does not state how rapid; if he had, we might form some conjecture of what he considers an "extremely rapid pulse and respiration." This visit must have been about six, or half-past six o'clock, and about two, or two and a half hours after I had left, at which time the pulse was precisely 132, respirations 44; resting quietly; had had no convulsion during the night; extremities warm; gently perspiring; no nausea or vomiting; could swallow readily; had taken veratrum regularly every three hours, without much trouble; had drank water freely, and yet he "could see no marked improvement" from her perilous condition the evening before, with pulse far too frequent to be counted, respirations 95, extreme restlessness and frequent convulsions, and requiring the almost constant use of ether and several attendants to keep her in bed. It is extremely gratifying to know that the half dozen faithful friends that remained with us that night, and all that saw her on these two occasions, (Dr. B. excepted,) could *not* "fail to see" a wonderful improvement. It will be remembered that I visited Ida again at ten o'clock, and found her symptoms all improved, "Dr. Boyce in attendance"—with "pulse 102, respirations 40—sweating freely," etc. Yet no improvement?

Again, he says: "As the day advanced, the patient became more quiet; the urine increased in quantity, with *frequent alvine evacuations.*"

It was stated in the report of this case, that a "small discharge" from the bowels had taken place in the after part of this day, and I will only refer to the certificate for additional reflections.

Query: if *one* insignificant evacuation could be thus construed "frequent alvine evacuations," would it not be at least methodical to rate a child's pulse beating steadily at 110 or less, as "extremely rapid"?

Again: "To these changes in the secretions I attribute the favorable change in *my* patient." Very likely; but, of course, veratrum, the only medicine she had taken for more .that 24 hours, had no agency in bringing about this "favorable change." We take no exception to his personal pronoun "*my*" patient, although I visited the patient with him in the morning and evening of this day.

But, again, he says: "If the patient took the veratrum under his directions, as stated in the *Journal*, then she got rather more than a double dose, and I cannot help but be thankful that *we* did not kill her outright."

Not to speak of the implication, (*vide* certificate), I concede that ten drops of veratrum, under ordinary circumstances, is a large dose for a patient of this age, but I think it a sound maxim that "desperate cases" sometimes "require desperate means," and the sequel proved that the dose was not too large; for all experience and all authorities agree that the remedy is entirely safe and harmless, when given to any extent, if below the nauseating point, and this was not reached in her case, and had it been, I presume the improvement would have been more marked, if possible. It has been my practice for several years to administer this remedy in emetic doses in membranous croup, and always with the happiest result, and I have never witnessed any dangerous effects resulting from its use.

For the benefit of the incredulous, let us briefly direct our attention to a few authorities that may not require a certificate to establish the veracity of their statements in regard to their experience with this drug; these are purposely taken from the records of several years' standing, and so far as I have been able to learn, the article has not only lost nothing of the reputation here referred too, by its recent and greatly extended use, but there is scarcely a well-informed physician at the present day who will not admit its great utility in the treatment of many of our worst forms of disease.

"It is reliable. As an arterial sedative, the Society has found it more certain than any other medicine of the class." "It is safe! The first indication of a sufficiently full use being nausea and diaphoresis, one or both."—Rep. of Middlesex Med. Soc., Mass.; Am. Med. Jour., 1858.

"I have rarely seen any unpleasant, certainly no dangerous symptoms from its use, and think it is not cumulative in its effects so as to need the close watching that digitalis requires. If the dose is too large, besides the slowness of the pulse always found, there will be nausea or vomiting and sweating. Should it be thought necessary to give a full dose of half a drachm to an adult at once, it can be done with perfect safety; at least, I have so used it."—B. Cutler, M.D., Am. Med. Jour., Oct. 1858.

H. P. Wakefield, M.D., states: "I have used veratrum viride as an arterial sedative, and have found it a reliable article." In every case he has found the pulse to come down. He deems the article far superior to digitalis, etc.—Am. Med. Jour., Oct. 1858.

E. Cutler, M.D., says: "I am satisfied that the veratrum viride is an arterial sedative, having used it as many if not more times than any other medicine."—Am. Med. Jour., 1858.

Dr. H. B. Toland, San Francisco, Cal., says: "Having administered it alone in one of the most painful and unmanageable of the curable diseases incident to the country, rheumatism, particularly in the acute stage, I have found it more efficacious than any remedy that has heretofore been employed; besides controling the action of the heart, it relieves pain, and is more decidedly diuretic than even colchicum."—Pac. Med. Jour., 1858.

Dr. G. M. Staples, Dubuque, Iowa, says his experience satisfies him that "we have in this drug an agent which enables the physician to maintain, for an indefinite time, complete control of the nervous and circulatory systems."—Med. & Surg. Rep., Phil., Aug. 3, 1861.

Dr. A. Hard, Aurora, Ill., has "found it the most certain of all arterial sedatives"—Chicago Examiner.

"We believe that few physicians who understand the virtue of veratrum viride are willing to dispense with its use."—Med. & Surg. Rep., Aug. 3, 1861.

We might multiply authorities to an almost unlimited extent, but I trust sufficient has been shown to convince the most skeptical "old fogy" that veratrum viride has some "claims that physicians are bound to respect."

Dr. B—— says: "The doctor made a mistake in the proper diagnosis. It was not, I think, a case of uræmia *per se*."

Who said it was? Is it not a little singular that the doctor in his "Criticism" commits the same mistake three several times by using the identical phraseology ("uræmic poisoning,") that the writer used in the published report of this case? I suppose that one at all conversant with medical literature, readily understands the meaning of this term in connection with, or succeeding a case of scarlatina; the merest tyro understands that the urea is not properly eliminated, but is retained, absorbed into the circula tion, inducing a fearful train of evils, which, if not speedily attended too, are dangerous in the extreme; in fact, it is regarded as the materies morbi in these cases.

Speaking of "mistake," just here I am reminded of a circumstance which, if does not indicate profound erudition in anatomy, verifies the adage that to "err is human"; it occurred in connection with a post mortem examination in this vicinity but a few months since, where an individual who always attaches an M.D. to his signature, *mistook the spleen* for a *kidney;* and that in a case which he had treated for several months as albuminuria, when the post mortem revealed a healthy *spleen*, one healthy kidney, (only one was examined,) and a vast amount of tubercular deposits in the mesentery and lungs; but for the credit of the fraternity, and the honor of medical schools, I will assure them he had never graduated.

He further says: "As to the question, 'Is it a poison?' it is too absurd to require comment."

By reference to our article, it will be noticed that I did not assume that it was or was not "a poison," but intimated that it was a "mooted question;" yet advoeated a discriminate use of veratrum viride from the stand-point that it was a poison, aye, a "virulent poison."

Let us for a moment examine this question. What constitutes a poison?

"That substance which, when applied externally, or taken into the human body, *uniformly* effects such a derangement in the animal economy as to produce disease."—Med. Dict.

"Any substance which, when applied externally, or in any way introduced into the system, without acting mechanically, but by its own inherent qualities, is capable of destroying life."—Dr. Guy—Elwell Mal. Prac. & Med. Evid., p. 441.

"A poison is commonly defined to be a substance which, when administered in *small quantities*, is capable of acting deleteriously on the body; and, in popular language, is confined to substances which destroy life in small doses."—Taylor on Poisons, p. 18.

I am yet to learn of a single instance of death clearly chargable to its account in all the years that it has been before the profession, while it has been used by thousands of our brethren from Maine to California. The nearest approach to it that I have seen, was that of a child 18 months old, who took, by mistake, 35 drops, and the account says, "It ultimately terminated fatally." Clearly intimating that it did not, like most cases of poisoning, prove directly fatal, if, indeed, it had anything to do with the result.—U. S. Disp., 13th Ed.

We scarcely pick up a medical journal without being startled with "Poisoning by Belladonna," by "Aconite," by "Hyoscyamus," "Digitalis," etc., etc., but who has read, or otherwise heard of a well-defined case of poisoning by veratrum viride, when taken by mistake, or otherwise, in small or large doses?

Dr. Pashley reports a case, where a man in health took, by mistake, *two ounces* of a saturated tincture at a draught, and speedily recovered.—N. W. Med. Jour., Nov. 1857.

Professor Barker, of Bellevue, gives it in ten drop doses, every hour, in typhoid fever.—Am. Med. Mon., Nov. 1857.

Ephraim Cutler, M.D., reports a case where his patient, "a young man, took from 20 to 30 drops at a dose, and was rapidly restored."—Am. Jour. Med. Sci., 1858, p. 313.

Dr. Norwood reports his case of "Daddy Billy," who took one teaspoonful of his (Norwood) tincture at a dose, and was well in a few hours. Vide Pamph. on V. V.

A. B. Clarke, M.D., relates a case of pneumonia, where, by mistake in the directions, his patient got "a teaspoonful every four hours, three large teaspoonsful in twelve hours," and he adds,

"the disease was broken up. He was very comfortable next morning. I saw him in the streets a day or two afterward." He further says: "Here was a case where the veratrum had reduced the pulse 100 per minute. Given by mistake in such large doses, it caused extreme depression and excessive vomiting, yet the patient readily rallied."—Med. & Surg. Rep., Phil., June, 1862.

What other poisonous substance, think you, this man could have taken in quantities six or eight times its maximum dose, with impunity? Would he have "appeared in the street in a day or two afterward" if he had taken like potions of belladonna, digitalis, aconite, or colchicum? Possibly, he might have done so, but he would doubtless have been encased in a wooden overcoat, accompanied by his friends marching to the solemn dirge of the tolling bell.

The doctor says, in conclusion: "That the vaunting of heroic remedies as specifics and cure-alls, savors of quackery," etc.

If this is designed to include the accurate and truthful reporting of cases, and the effects of remedies therein, at which, and only which, this "Criticism" was aimed, then I must plead guilty to the charge; but it is hoped that the great body of intelligent physicians, who are wont to do so, will not cease to occasionally report important and interesting cases and their treatment, notwithstanding.

"Savors of *quackery!*" This inauspicious word of three syllables, how easily written! but to me it has always seemed that it should ever be softly spoken by those who have never availed themselves of any of the advantages so cheaply afforded by our numerous medical colleges and schools for acquiring a sound and systematic medical education.

In conclusion, I will add that I do not regard veratrum viride a "specific," or entirely harmless when administered improperly, regardless of circumstances; but I do contend, that, from the vast array of facts, together with our own experience, it is not a doubtful or dangerous remedy, but is perfectly reliable when given under proper circumstances, and in proper doses, and, with the ordinary care required in the administration of most medicines, that it will seldom, if ever, fail of producing its characteristic effects on the circulation. I believe, also, that it has an important though secondary sedative effect on the *cerebro-spinal* nervous system, and when full and efficient doses are administered, it induces decided sedation of the great sympathetic nervous system. To my mind, it is these properties which render it one of the most certain and reliable remedies known to the Materia Medica in just such cases and conditions as the one which has called out this acrimonious "Criticism."

